# Change in the Structure of *Escherichia coli* Population and the Pattern of Virulence Genes along a Rural Aquatic Continuum

**DOI:** 10.3389/fmicb.2017.00609

**Published:** 2017-04-18

**Authors:** Fabienne Petit, Olivier Clermont, Sabine Delannoy, Pierre Servais, Michèle Gourmelon, Patrick Fach, Kenny Oberlé, Matthieu Fournier, Erick Denamur, Thierry Berthe

**Affiliations:** ^1^Normandie Université, UniRouen, UniCaen, CNRS UMR M2CRouen, France; ^2^Sorbonne Universités, UPMC, CNRS, EPHE, UMR 7619 METISParis, France; ^3^INSERM UMR1137, IAME, Université Paris Diderot, Sorbonne Paris CitéParis, France; ^4^Université Paris-Est, Anses, Food Safety Laboratory, IdentyPath Platform, Maisons-AlfortFrance; ^5^Ecologie des Systèmes Aquatiques, Université Libre de Bruxelles, Campus de la PlaineBruxelles, Belgium; ^6^Institut Français de Recherche pour l’Exploitation de la Mer, RBE-SG2M-LSEMPlouzané, France

**Keywords:** water, sediment, pathogenic *E. coli*, virulence gene, Shiga toxin

## Abstract

The aim of this study was to investigate the diversity of the *Escherichia coli* population, focusing on the occurrence of pathogenic *E. coli*, in surface water draining a rural catchment. Two sampling campaigns were carried out in similar hydrological conditions (wet period, low flow) along a river continuum, characterized by two opposite density gradients of animals (cattle and wild animals) and human populations. While the abundance of *E. coli* slightly increased along the river continuum, the abundance of both human and ruminant-associated *Bacteroidales* markers, as well as the number of *E. coli* multi-resistant to antibiotics, evidenced a fecal contamination originating from animals at upstream rural sites, and from humans at downstream urban sites. A strong spatial modification of the structure of the *E. coli* population was observed. At the upstream site close to a forest, a higher abundance of the B2 phylogroup and *Escherichia* clade strains were observed. At the pasture upstream site, a greater proportion of both E and B1 phylogroups was detected, therefore suggesting a fecal contamination of mainly bovine origin. Conversely, in downstream urban sites, A, D, and F phylogroups were more abundant. To assess the occurrence of intestinal pathogenic strains, virulence factors [*afaD, stx1, stx2, eltB* (LT), *estA* (ST), *ipaH, bfpA, eae, aaiC* and *aatA*] were screened among 651 *E. coli* isolates. Intestinal pathogenic strains STEC O174:H21 (*stx*2) and EHEC O26:H11 (*eae, stx*1) were isolated in water and sediments close to the pasture site. In contrast, in the downstream urban site aEPEC/EAEC and DAEC of human origin, as well as extra-intestinal pathogenic *E. coli* belonging to clonal group A of D phylogroup, were sampled. Even if the estimated input of STEC (Shiga toxin-producing *E. coli*) *–* released in water at the upstream pasture site – at the downstream site was low, we show that STEC could persist in sediment. These results show that, the run-off of small cattle farms contributed, as much as the wastewater effluent, in the dissemination of pathogenic *E. coli* in both water and sediments, even if the microbiological quality of the water was good or to average quality according to the French water index.

## Introduction

In the upcoming decades, the vulnerability of environmental water to contamination by fecal pathogens will become an increasingly major public health concern. This is due to the expected increase in the human population, the related agricultural activities, as well as climate change responsible for more frequent flood events (Mallin et al., 2000; Hales and Corvalan, 2006; Bartram and Cairncross, 2010). To assess the microbiological quality of environmental water, *Escherichia coli* has been chosen as one of the two bacterial indicators of fecal contamination, according to the World Health Organization and European regulations (2006/7/EC, WHO, 2011).

However, high genetic and phenotypic diversities exist within the *E. coli* population, which could be divided into seven major phylogroups, to which *E. coli* clades have been added (Gordon, 2010; Tenaillon et al., 2010). While *E. coli* are the most abundant aerobic culturable bacteria in the microbiota gut of humans and animals, some *E. coli* strains are also important human pathogens (Kaper et al., 2004). Among the eight pathovars identified, six diarrheagenic pathovars implicated in waterborne outbreaks have been characterized. These include enteropathogenic *E. coli* (EPEC), enterotoxigenic *E. coli* (ETEC), enteroinvasive *E. coli* (EIEC), enteroaggregative *E. coli* (EAEC), diffusely adherent *E. coli* (DAEC), and enterohemorrhagic *E. coli* (EHEC) (Croxen and Finlay, 2009). These intestinal pathogenic *E. coli* have been thoroughly characterized by a set of specific virulence genes which can be used as molecular targets to detect their presence in water or sediment (Sidhu et al., 2013; Haack et al., 2015).

Today, there is an increasing interest in EHECs, the zoonotic Shiga toxin-producing *E. coli* (STEC), which can cause severe diarrheas, hemorrhagic colitis, sometimes associated with hemolytic uremic syndrome (Williams et al., 2012). The contamination of water by Shiga toxin-producing *E. coli* (STEC/EHEC), mainly *E. coli* O157:H7 but also non-O157 STEC, have resulted in numerous outbreaks associated with both recreational and drinking water (Saxena et al., 2015). In France, the main STEC associated with severe gastroenteritis, related to food or water consumption, belong to the five highly pathogenic serotypes O157:H7; O26:H11, O111:H8, O103:H2, and O145:H28 and more recently O80:H2 (INVS, 2007; ANSES, 2010; Delannoy et al., 2015; Soysal et al., 2016).

Moreover, widespread of antibiotic-resistant *E. coli* in water, mainly strains harboring integrons, is also a major public health concern (Stalder et al., 2012). Indeed, the proportion of *E. coli* harboring class I integron reached 21% in beach water, and ranged between 21 and 11% in estuary water or between 0 and 11% in karst aquifer depending on the hydrological conditions (Flores Ribeiro et al., 2012; Lupo et al., 2012; Moura et al., 2014; Ghaderpour et al., 2015). Indeed, clinical integrons, considered to be xenogenetic contaminants, could be employed as bioindicators of the risk of the spread of antibiotic resistance in the environment (Gillings, 2014; Borruso et al., 2016).

In surface water, both the diversity of the *E. coli* population and the occurrence of pathogenic *E. coli* are related to the anthropogenic pressure – i.e., human or animal density – exerted on the watershed combined with the hydrologic conditions (Crowther et al., 2002; DiDonato et al., 2009; Viau et al., 2011; Sidhu et al., 2013). Once released in water, the population structure of *E. coli* can be modified: it reflects both their primary host and their fate in this environment (Berthe et al., 2013; Chandran and Mazumder, 2015). In environmental water, spatial and seasonal changes of the *E. coli* population diversity have been demonstrated using fingerprinting methods (Chandran and Mazumder, 2015). The distinct ability to overcome environmental stress leads to the selection of *E. coli* strains exhibiting better survival in the environment. Some of these strains have been considered as naturalized in soil, sediment, or water (Kon et al., 2007; Garzio-Hadzick et al., 2010; Bergholz et al., 2011).

Intra-intestinal pathovars have been detected within the *E. coli* population in water impacted by the discharge of wastewater treatment plants (WWTPs) (Sidhu et al., 2013; Yang et al., 2014) or contaminated by the runoff water from large agricultural areas, or discharges from mixed land-use watershed (Balière et al., 2015; Gomi et al., 2015). However, within these intra-intestinal pathovars, a very low frequency (0.2%) or no STEC were observed in treated effluent of WWTPs or slaughterhouses (Diallo et al., 2013; Yang et al., 2014; Um et al., 2016). Higher prevalence of EHEC (i.e., presence of *stx2* and *eae* genes) has been reported in lake water in Canada, corresponding to 1.8% (4/658 isolated *E. coli*) (Chandran and Mazumder, 2015). In France, prevalence of STEC in freshwater or seawater reached 0.17% (14/8,371 isolated *E. coli*), and 0.44% (1/225), respectively, without isolation of a EHEC strain (Balière et al., 2015).

In France, Normandy is a region with a high surface area devoted to agricultural activities and where the climate is oceanic with abundant rainfall throughout the year. In this area, permanent grassland accounts for 70% of the total crop surface with around 14,700 cattle farms, averaging 50 cattle per farm^1^. Three main outbreaks associated with STEC/EHEC have been there reported (O26:H11, O80:H2, and O157:H^-^), due to the consumption of cheese made with raw milk (INVS, 2007; ANSES, 2010). The main reservoir of STEC/EHEC being the digestive tract of cattle, the runoff on pastured lands is associated to the contamination of environmental water by these highly pathogenic *E. coli* (Chandran and Mazumder, 2015; Saxena et al., 2015). Thus, water contaminated by STEC/EHEC is a pathway for the dissemination of these highly pathogenic *E. coli* between the three main reservoirs: animals, water, and humans. However, to date, the prevalence of pathogenic strains released in water by such small cattle farms has been poorly documented.

The aim of this study was to investigate the structure of the *E. coli* population, based on their phylogroup, their antibiotic resistance profiles, and the prevalence of intestinal pathogenic strains along rivers draining rural watershed (123 km^2^), in which cattle farming activities are representative of practices in France. For this purpose, monitoring of waterbodies was carried out during two sampling campaigns with similar hydrological conditions (wet period during a low flow period) along a river continuum (8 km), characterized by two opposite density gradients of animals (cattle and wild animals) and human populations. Finally, we estimated the putative input of STEC/EHEC released in a stream at the upstream part of the network to the downstream river.

## Materials and Methods

### Study Site and Sampling Strategy

**FIGURE 1:**
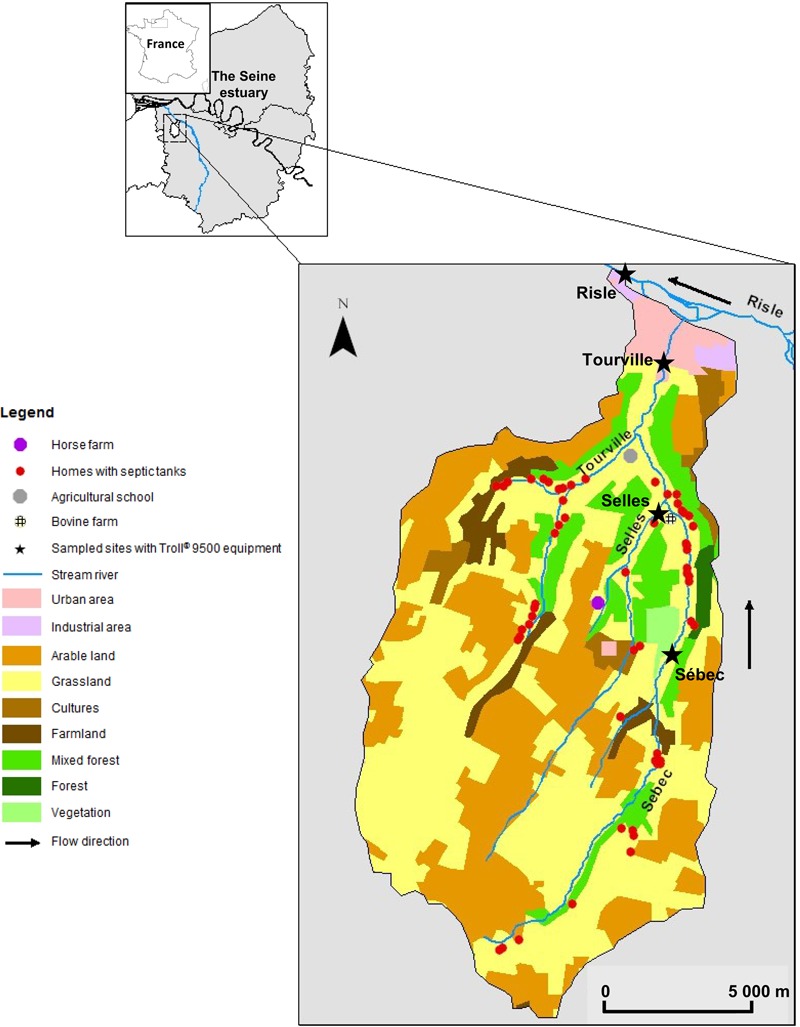
**Map of the studied watershed with land use following Corine Land Cover Database**.

The study site (123 km^2^) is a rural aquatic continuum located in northwestern France (Western Europe). It is part of the Seine River hydrographical network (**Figure 1**). The climate in this area is oceanic with 678.4 mm of rainfall during the hydrological year (2009–2010). Monthly rainfall ranges from 16.0 to 134.4 mm with high rainfall events during the winter season. The aquatic continuum studied is composed of four rivers (Selles, Sébec, Tourville, and Risle). The Selles flows into the Sébec River, which then flows into the Tourville River. Finally, the Tourville River flows into the Risle River, the main tributary in the mouth of the Seine estuary (**Figure 1**). Land cover of the stream watersheds was determined using ArcGIS 10.1 (ESRI, Redlands, CA, USA). The land use of the three upstream sub-watersheds (altitude around 150 m) is dominated by grasslands (pastures account for 48% of the watershed surface at Tourville) and arable areas, on which manure can be spread (35% of the watershed surface at Tourville) while forests account for only 4% of the watershed surface at Tourville. High animal density and a low human population also characterize these rural watersheds. In contrast, urban areas (3.2% of the total surface of the Risle watershed) were predominant at the downstream site (altitude 25 m) with a higher human density (11,342 inhabitants) without cattle. The efficiency of domestic wastewater treatment increases from upstream, where septic tanks are mainly used, to downstream, where a WWTP treats the wastewater of the urbanized zone (29,161 inhabitant equivalents) (**Table 1**).

**Table 1:** Description of studied sites.

			Selles	Sébec	Tourville	Risle (Pont audemer)
**Hydrological parameters^a^**	Geomorphological river order		1	2	3	4
	Mean velocity (ms^-1^)		0.1	0.1	0.2–0.3	0.5-1
	Mean waterflow^a^ (m^3^ s^1^) Range		0.05 (0.05–0.6)	0.05 (0.01–0.6)	0.34 (0.1–2)	11.8 (6.6–115)
	Waterbodies Transit time (h)^b^	Rain event		3.5^d^		2.5^e^
		Dry period		12^d^		10^e^
	Dilution rate^c^	Rain event		1:3^d^		1:20^e^
		Dry period		1:6^d^		1:40^e^
**Surface and land use of**	Basin surface (km^2^)		7.61	14.94	53.87	2153.67
**watershed (%)**	Urban and industrial areas		0.0	0.0	0.0	3.0
	Arable		47.0	33.6	34.7	50.6
	Grassland		35.0	56.0	47.5	24.2
	Farm land		5.1	2.8	6.2	4.0
	Forest		12.9	7.6	4.0	18.3
**Physico-chemical**	Mean SPM^a,f^ (mg L^-1^) Range		NA	9.0 5–21	6.4 2.0–37	19 10–30
**parameters**	Mean DOC^a,f^ (mg L^-1^) Range		NA	1.7 1.1–4.3	2.2 0.8–6.2	2 1.3–3.7
	Mean temperature^a,f^ (°C) Range		NA	11 6–14	11 8.5–14	11.3 10–19
**Human and animal**	Inhabitants^g^		396	278	2284	166 340^h^
	Wild animals Range *N* ha^-1^ (boar /roe/deer)^i^		40–50	54–150 (1–250)	NA	NA
	Head of cattle (*N*)^j^		719 (50 ± 10)	200	57	NA
**Fecal bacteria inputs**	Distance from the closest pasture land (run-off)		50 m	>1 km	NA	NA
	Septic tanks^k^ (malfunctioning tanks)		159 (42)	92	168 (45)	97 (29)^l^

*^a^Dataset (data set from the French Water Agency, Seine Normandy); ^b^Transit times correspond to the time to go to the upstream site from the downstream site; ^c^Dilution rates correspond to the ratio discharge between downstream and upstream sites; ^d^transit time from Selles site to Risle site; ^e^transit time from Tourville site to Risle site; ^f^annual mean; ^g^cumulative sum of human densities from upstream to the sampling site; ^h^upstream of the sampling site, the Risle River drains a watershed with 155,000 inhabitants and flows through an urbanized zone with 11,342 inhabitants; ^i^data from ONCSF 2010; ^j^head of cattle on pasture closest to the sampling site; ^k^on the run-off watershed; ^l^located in the Pont Audemer urban area; SPM, suspended particulate matter; DOC, dissolved organic carbon; NA, not available.*

Along this water system, four sampling sites were defined according to the water vulnerability to microbial contamination. At the upstream part, two sampled sites were located in the Selles River (order-one river) and in Sébec River (order-two river); they were mainly impacted by bovine contamination. The first sampled site from the Sébec River is located close to a forest area (presence of boars, roes and deers) and 4 km downstream from several dairy farms (around 200 cows). The second sampled site is located on the Selles River, in the immediate vicinity of a farm (50 m) with 50–60 cattles. The third sampled site, in the Tourville River (order 3), downstream of its confluence with the Sébec, was assumed to be impacted by both human and bovine contamination. A school (250 ± 30 persons) with malfunctioning septic tanks is located 1 km upstream from this sampling point. The fourth sampling site, is the Risle River (order 4), downstream of the confluence of the Risle and the Tourville rivers, is located in an urban area (11,342 inhabitants). The population of this urban area is connected to a WWTP, which releases its treated effluents into the Risle River downstream from our sampling station, except for 97 household septic systems.

**FIGURE 2:**
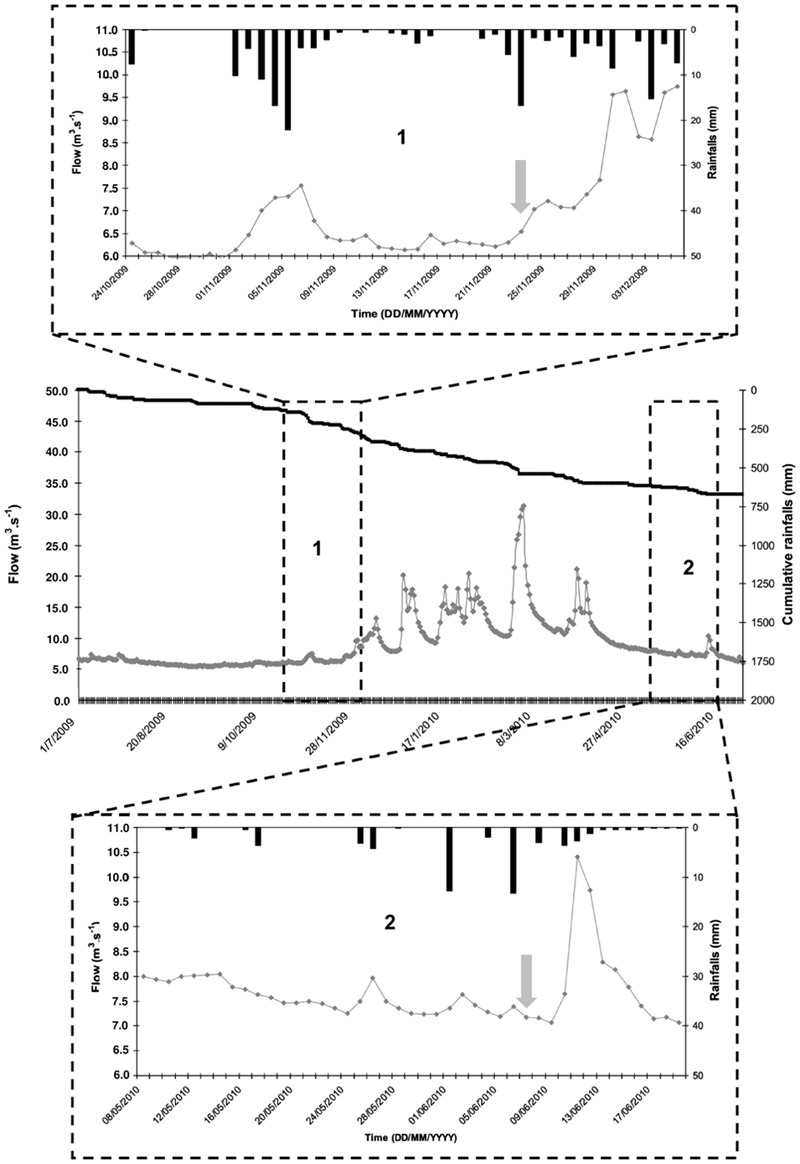
**Hydrological conditions of the study period: flow of the Risle River measured at hydrometric station located at 15 km in upstream of the Risle sampling site (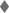), cumulative rainfall (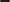), and rainfall (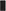) during the hydrological year.** The two sampling campaigns (vertical grey-arrows) were carried out in similar hydrological conditions in November 2009 (1) and June 2010 (2). These hydrological conditions represent the average hydrometeorological context of the region studied, according to flow and rainfall (around 55% of flows and rainfall cumulative distribution functions).

Two sampling campaigns were carried out during the fall season (November 2009) with cow stalling, and during the spring season with cattle grazing (June 2010). The hydrological conditions were comparable, with similar amounts of precipitation (10 and 15 mm) followed by very small changes in discharge during a low flow period in Risle River (≈ 6.5 m^3^.s^-1^ at hydrometric station located 15 km away from the upstream of the Risle sampling site), as shown in **Figure 2**. These hydrological conditions represent the average hydro-meteorological context of the region studied, according to flow and rainfall (around 55% of flows and rainfall cumulative distribution functions, Meteo France Database^2^). Water and sediment samples were collected the same day from upstream to downstream. Water samples were collected at each station of the continuum using auto-samplers (1 L collected every hour for 24 h). In order to monitor the waterbodies, the starting time of the auto-sampling was chosen taking the flow rate of the rivers into account. Superficial sediments (0–1 cm depth) were sampled, using a sterile sub-corer (50-mL sterile plastic tube; Corning), on the temporary deposit areas of particles close to (±1 m) the four sampling sites. In June 2010, no sediment deposit areas were observed near the Selles sampling site. Water and sediment samples were stored at 4–6°C and transported to the laboratory within 3 h. Once in the laboratory, 250 mL from each flask of the auto-samplers were pooled to obtain a 24-h average sample for analysis. Microbiological analyses were carried out within 8 h.

### Chemical and Physical Parameters

Temperature, water level, turbidity, and pressure were measured every 15 min using Troll^®^ 9500 *in situ* Water Quality instrument (In-Situ Inc.). An ISCO 674 tipping bucket rain gauge (Teledyne ISCO Inc.) in the Selles sampling site was used to record rainfall. To determine the suspended particulate matter (SPM) concentration, 100 mL of water was filtered through pre-weighed 0.45-mm pore-size filters (Millipore) that were dried for 48 h at 50°C before being weighed again to determine the total SPM concentration (NF T90-105-2, ISO11923).

### Enumeration and Isolation of Culturable *E. coli* and *Enterococcus*

*Escherichia coli* and *Enterococcus* were enumerated using membrane filtration methods (0.45 μm HA047, Millipore). β-D-galactosidase-positive and β-D-glucuronidase-positive *E. coli* were isolated from the water samples with selective chromogenic media specific for *E. coli*, with the addition of a selective supplement for water samples (RAPID’*E. coli* 2 Medium and Supplement; Biorad). Plates were incubated for 24 h at 37°C. *Enterococcus* was isolated from the water samples with selective chromogenic media specific for *Enterococcus* (RAPID’*Enterococcus* Medium; Biorad). Plates were incubated for 48 h at 44°C. Sediment was analyzed with the following modifications: 2 g (wet weight) were added to 18 mL of NaCl 0.85% (w/v) supplemented with Na_4_P_2_O_7_ (1 mM, final concentration) and mixed vigorously for 3 min to dissociate bacteria from organic mineral particles (Berthe et al., 2008). Ten-milliliter volumes of appropriate dilutions were then filtered before plating. The threshold values for the enumeration of *Enterococcus* and *E. coli* in water was 5 CFU 100 mL^-1^. For each site sampled, about 50 non-confluent colonies of *E. coli* were randomly selected on the filter and then streaked on Luria Broth agar (Gibco). Finally, a total of 651 *E. coli* strains (β-D-galactosidase-positive and β-D-glucuronidase-positive) were thus isolated and stored on a CryoBeads^®^cryo-bead system (Biomérieux) at -80°C. The loss of culturability of pathogenic *E. coli* strains was monitored over 14 days at 10°C in an estuarine filtered-water microcosm under dark conditions as described by Berthe et al. (2013).

### Antibiotic Resistance Testing of *E. coli*

*Escherichia coli* resistance to antibiotics was tested using the agar diffusion method according to the recommendations of the Comité de l’Antibiogramme de la Société Française de Microbiologie (CA-SFM^3^). *E. coli* CIP 7624 (ATCC 25922) was used as a control. The tested antibiotics (17) included the most commonly used in France for the treatments of *E. coli* infections in human and veterinary medicine: amoxicillin (AMX, 25 μg), amoxicillin + clavulanic acid (AMC, 20 + 10 μg), ticarcillin (TIC, 25 μg), ticarcillin + clavulanic acid (TIM, 75 + 10 μg), imipenem (IPM, 30 μg), cephalothin (CEF, 30 μg), ceftazidime (CAZ, 30 μg), cefotaxime (CTX, 30 μg), gentamicin (GEN, 15 μg), kanamycin (KAN, 30 IU), streptomycin (STR, 10 μg), chloramphenicol (CHL, 30 μg), tetracycline (TET, 30 μg), trimethoprim-sulfamethoxazol (SXT, 23.75 + 1.25 μg), nalidixic acid (NAL, 30 μg), ciprofloxacin (CIP, 30 μg), and chloramphenicol (C, 30 μg). As recommended by Magiorakos et al. (2012), *E. coli* strains resistant to at least one antibiotic in three or more antimicrobial classes were considered as multi-resistant.

### Genomic Characterization of the *E. coli* Strains

The phylogenetic group of the *E. coli* isolates was determined using the PCR-based method, as proposed by Clermont et al. (2013). The identification of *Escherichia* clade strains, which are phenotypically undistinguishable from the *E. coli sensu stricto* strains, were performed by PCR as described in Clermont et al. (2011). The *E. coli* B2 group strains were classified into subgroups by an allele-specific PCR assay as described in Clermont et al. (2014). The characterization at the clone level defined as the association of the clonal complex with the O-type was carried out as previously described by (Bidet et al., 2007). The clonal group A (CGA) among the D phylogroup strains was identified by PCR (Johnson et al., 2004). The presence of virulence factors involved in intra-intestinal pathogenesis [*afaD, ipaH stx1, stx2, eltB* (LT), *estA* (ST), *bfpA, eae, aaiC* and *aatA*] was detected using conventional PCR, as previously described by Escobar-Paramo et al. (2004). The O-type of intestinal pathogenic and B2 strains was determined by an allele-specific PCR, as previously described (Clermont et al., 2007). Multilocus sequence typing (MLST) was performed on the intestinal pathogenic strains using the Pasteur Institute scheme (Clermont et al., 2015). Molecular detection of the class 1 integrase gene was carried out by PCR in *E. coli* isolates, with the specific primers intI1.F/intI1.R, as previously described (Bass et al., 1999). Clone relatedness was assessed by random amplified polymorphic DNA (RAPD) using primer and conditions as described in (Clermont et al., 2013).

In addition to conventional PCR assays, a qPCR microarray has been designed on the LightCycler 1536 (Roche Diagnostics, Meylan, France) to screen the isolates for *E. coli* virulence or characteristic genetic markers that were selected according to their role in pathogenesis, their ability to be associated with human and non-human animal illness and because they had previously been shown to be useful for the characterization of STEC (*stx1, stx2*), EPEC (*eae*) and EAEC (*aggR, aggA-I, aggA-III, aatA, aap, pic, set1*) strains (Delannoy et al., 2012). The genetic markers tested are many type III effector genes, toxin-producing genes or adhesin-producing genes present in the following pathogenicity islands: HPI (*irp2, fyuA*), OI-15 (*ehaA*), OI-43 (*Z1151, Z1153, Z1155, Z1156*), OI-43/48 (*iha, terE, ureD*), OI-44 (*espV*), OI-50 (*espK, espN, espX7, espO1-1*), OI-57 (*Z2096, Z2098, Z2099, Z2121, ecs1763*), OI-71 (*nleF, nleG, espM1, nleH1-2, nleA, Z6065, ecs1822*), OI-108 (*espM2, espW*), OI-122 (*pagC, ent, nleB, nleE, Z4331, efa1, efa2*), OI-174 (*espX6*), in plasmids, e.g., pO157 (*ehxA, ecf1, toxB, katP, etpD, espP*), pO113 (*epeA, saa, sab, subA*) or in the chromosome. Genetic markers related to serogroups (*wzx*_O174_, *wzx*_O81_, *wzx*_O26_, *wbd*_O111_, *wzy*_O153_), flagellar antigens (*fliC*_H1/H12_, *fliC*_H2_, *fliC*_H8_, *fliC*_H21_, *fliC*_H25_, *fliC*_H11_), long polar fimbriae (*lpfA*_O113_, *lpfA*_O157_, *lpfA*_O26_), fimbrial adhesion (*F18*), bundle forming pilus (*bfpA*), alpha-hemolysin (*hlyA*), cytolethal distending toxin (*cdt-V*), heat stable enterotoxin (*astA*) or antimicrobial resistance (*blaCTX-M15, blaTEM1*) were also included. Most of the primers and probes used on the array have been described previously. *Stx*-typing (Stx1, Stx2) was performed using primers and probes described by Perelle et al. (2004). Subtyping of the *eae* genes (*eae*-gamma, *eae*-beta, *eae*-epsilon, *eae*-theta) was performed according to Nielsen and Andersen (2003). The *wecA* gene, which is part of the *wec* cluster that codes for the synthesis of the enterobacterial common antigen, was used as a reference marker for *E. coli* (Bugarel et al., 2011). Further serogroup determination was performed by next generation sequencing of the O-antigen gene cluster essentially as described by Iguchi et al. (2015). The additional *fliC* gene sequences were determined by PCR and Sanger sequencing (Beutin et al., 2015).

### Detection of General and Host-associated *Bacteroidales* 16S rRNA Gene Marker

DNA was extracted from 500 mL of filtered water (0.45 μm HVLP047; Durapore, Millipore) using the Fast DNA for soil kit (MP Biomedical, Illkirch, France), with an extra washing step with the salt/ethanol wash solution (SEWS-M). General (AllBac), human (HF183), and ruminant (Rum-2-Bac)-associated *Bacteroidales* MST markers were quantified using real-time PCR following Mauffret et al. (2012) and using primers and probes described by Layton et al. (2006) and Mieszkin et al. (2010), respectively. For each MST marker (AllBac, HF183, and Rum-2-Bac), three dilutions of each DNA extract were analyzed (non diluted, 10- and 100-fold diluted). Furthermore, presence of PCR inhibitors was assessed using an Internal Positive Control (TaqMan Exogenous IPC reagents kit; Applied Biosystems, France) as an additional PCR reaction in the AllBac assays. When PCR inhibitors were present, concentrations of MST markers obtained in DNA extracts diluted 10- or 100-fold were retained to prevent the inhibitors from influencing the subsequent host-associated markers PCR reaction. Linear DNA plasmids containing partial 16S rRNA gene sequence inserts were used as standards at 10-fold dilutions ranging from 1.6 10^7^ to 1.6 10^0^ copies per PCR, with a quantification limit of five target gene copies/reaction per PCR well in the triplicate PCR assays. It was the lowest quantity of linear plasmid DNA of the standard curve made for each PCR assay. Correlation coefficients (r2) for all the standard curves were >0.97 and PCR efficiency ranged between 95 and 105%.

### Statistical Analysis

The percentages of *E. coli* phylogroups were compared (i) from upstream to downstream of the watershed and (ii) for each site between the two sampling periods using the chi-square test. Percentages of antibiotic-resistant *E. coli* were also compared from upstream to downstream using the same test. Tests were carried out using the XL Stats version 6.0 (Addinsoft).

## Results

### Origin and Abundance of Contamination by *E. coli* and *Enterococcus*

Both sampling campaigns were conducted in similar hydrological conditions. Runoffs were mainly responsible for contaminant inputs in surface water and contributed to an increase of the Risle river flow, final outlet of the watershed (**Figure 2**). The density of *E. coli* slightly increased in water from the upstream rural site (Sébec) to the downstream urban site (Risle) (respectively, 9 ± 2 × 10^2^ CFU 100 mL^-1^ and 9.9 ± 4.1 × 10^3^ CFU 100 mL^-1^). In contrast, no change in *Enterococcus* density was observed (**Table 2**). The *E. coli*/*Enterococcus* ratio increased along the continuum from 0.3 to 0.6 at the two upstream sites (Sébec and Selles) to 2.4 at the downstream urban site (Risle). The molecular quantification of human (HF183)- and ruminant (Rum2Bac)- associated *Bacteroidales* markers showed that, at both upstream sites, the microbiological contamination of water was mainly from ruminants while, at downstream urban site (Risle), the contamination originated mainly from humans (**Table 2**).

**Table 2:** Abundance of bacterial indicators of fecal contamination and *Bacteroidales* markers along a rural water continuum.

	Selles	Sébec	Tourville	Risle
**Fecal contamination**				
(CFU 100 mL^1^)				
*Escherichia coli*	1.6 ± 0.3 10^3^	9 ± 2 10^2^	2.1 ± 0.7 10^3^	9.9 ± 4.1 10^3^
*Enterococcus* sp.	4.9 ± 4.8 10^3^	1.5 ± 1.5 10^3^	1.2 ± 0.9 10^3^	4.1 ± 3.2 10^3^
***Bacteroidales* markers**				
Log_10_ (number of copies 100 mL^-1^)				
AllBac	6.8 ± 0.2	6.45 ± 0.05	6.75 ± 0.15	6.9 ± 0.1
HF183	ND	ND or <LQ	4.0 ± 0.2	5.1 ± 0.1
Rum2Bac	5.2 ± 0.5	5.0 ± 0.1	4.6 ± 0.5	<LQ

*ND, Not detectable by qPCR analysis; <LQ (limit of quantification): detectable by PCR analysis but below the limit for quantification by qPCR.*

### Change in *E. coli* Population Structure and Antibiotic Resistance Pattern

**FIGURE 3:**
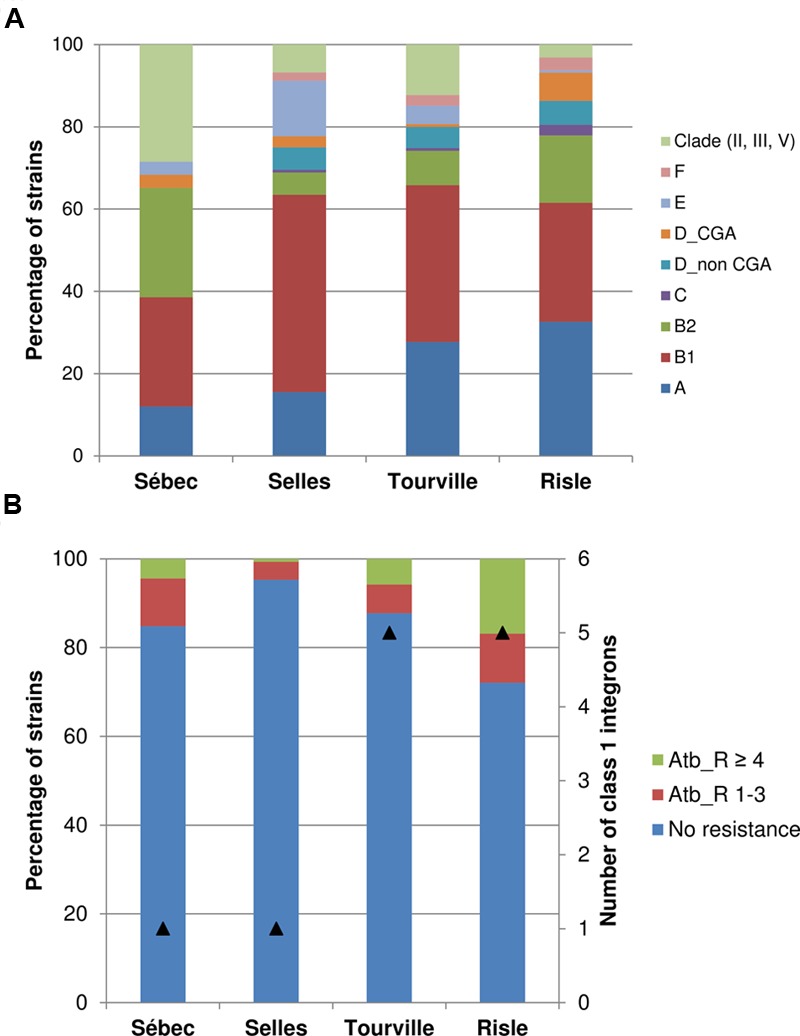
**Phylogroup distribution (A)** and antibiotic resistance **(B)** of *E. coli* populations along a rural continuum. The numbers of analysed strains were *n* = 158, *n* = 148, *n* = 155, and *n* = 190 for Sébec, Selles, Tourville and Risle respectively. The symbol (▲) corresponds to the number of class 1 integrons (*intI1*) identified among the strains. CGA, clonal group A; Atb_R 1–3, strains resistant from one to three antibiotics; Atb_R ≥ 4, strains resistant to at least 4 antibiotics.

The structure of the *E. coli* population in water did not significantly differ from the structure of the *E. coli* population from sediments for each sampling site. However, a change of the phylogroup distribution within the *E. coli* population from both sediment and water was observed along the continuum (**Figure 3** and Supplementary Table S3). In the upstream part, at the pasture site (Selles), both E and B1 phylogroups were significantly more abundant (*p*-value < 0.001) than at the other sampling sites. A significantly higher abundance of the B2 phylogroup and *Escherichia* clade were observed at the site located close to a forest (Sébec) than at the other sites. At the Tourville and Risle sites (urban areas), phylogroup A was significantly more abundant than in the upstream rural sites (Selles and Sébec). A significant increase of both D and F phylogroups (*p*-value < 0.001) was also observed at the urban Risle site. Within the phylogroup D strains, the uropathogenic *E. coli* clonal CGA was mainly isolated in water sampled in the downstream urban site (Risle).

A further analysis of the distribution of the B2 strains in subgroups (or clonal complexes) by an allele-specific PCR assay – combined with the determination of the most frequently O-types encountered in this phylogroup – were carried out to refine the epidemiology. The characterization at the clone level was defined as the association of the clonal complex with the O-type. It showed that 50% (21/42) of the B2 strains from the site close to a forest (Sébec) were unassignable-O non-typable, while typical ExPEC human clones belonging to subgroups I (STc131-O25b), II (STc73-O18), III (STc127-O6), VI (STc12-O4) and IX (STc95-O1, STc95-O18) were found only at the downstream urban site (Risle) (Supplementary Table S1; Clermont et al., 2014; Riley, 2014; Day et al., 2016). The proportion of *E. coli* isolates resistant to at least one antibiotic – among the 17 antibiotics tested – ranged from 5% (7/148) at the upstream pasture site (Selles) to 27% (53/190) at the downstream urban site (Risle). However, at the upstream site (Sébec) located close to a forest area (presence of boars and deers) 4 km downstream from several dairy farms, 15% (24/158) of *E. coli* isolates were resistant to at least one antibiotic. Analysis of multi-resistant *E. coli* isolates showed a significant increase (*p*-value < 0.001) from the upstream pasture site (Selles) to the downstream site (Risle). The occurrence of *E. coli* harboring class 1 integron was higher downstream (on average 5.0%) than upstream (on average 1.0%) in the continuum studied (**Table 3**).

**Table 3:** Main characteristics of the intestinal pathogenic *E. coli* isolated along the continuum.

Site		Strain	Phylo-group (subgroup)	ST^a^	Serotype	Pathovar^b^	*afaD*	*ipaH*	*eltB* LT	*estA* ST	*aatA*	*aaiC*	*eae* (type)	*bfpA*	*stx1* (subtype)	*stx2* (subtype)
Selles	Water	EC4199^c^	B1	501	O174:H21	STEC	–	–	–	–	–	–	–	–	–	+ (d)
		EC6086^c^	B1	501	O174:H21	STEC	–	–	–	–	–	–	–	–	–	+ (d)
	Sediment	EC6089	B1	481	O26:H11	EHEC	–	–	–	–	–	–	+ (beta)	–	+ (a)	–
		EC6096	B1	481	O26:H11	EHEC	–	–	–	–	–	–	+ (beta)	–	+ (a)	–
		EC6111	B1	481	O26:H11	EHEC	–	–	–	–	–	–	+ (beta)	–	+ (a)	–
		EC6115	B1	481	O26:H11	EHEC	–	–	–	–	–	–	+ (beta)	–	+ (a)	–
Tourville	Water	EC6230	E	673	O153:H31	aEPEC	–	–	–	–	–	–	+ (theta)	–	–	–
	Sediment	EC6316	E	673	O153:H31	aEPEC	–	–	–	–	–	–	+ (theta)	–	–	–
Risle	Water	EC4312	A	674	O99:H10	EAEC	–	–	–	–	+	+	–	–	–	–
		EC4321	A	750	O10:H4	DAEC	+	–	–	–	–	–	–	–	–	–
		EC4330	B2 (UA^d^)	751	O81:H6	aEPEC	–	–	–	–	–	–	+ (beta)	–	–	–
		EC6138	B1	696	O88:H25	EPEC	–	–	–	–	–	–	+ (epsilon)	+	–	–
	Sediment	EC4342	B1	752	O111:H21	EAEC	–	–	–	–	+	+	–	–	–	–

*STEC, Shiga toxin-producing *E. coli*; EHEC, enterohemorrhagic *E. coli*; aEPEC, atypical enteropathogenic *E. coli*; EAEC, enteroaggregative *E. coli*; DAEC, diffusely adherent *E. coli*. ^a^According to the Pasteur Institute scheme (Clermont et al., 2015). ^b^According to Nguyen et al. (2006). ^c^Two different sampling periods. ^d^ UA: unassignable according to Clermont et al. (2014).*

### Occurrence of Intestinal Pathogenic Strains of *E. coli*

To assess the occurrence of intestinal pathogenic strains in water and sediments along the continuum, 10 virulence factors were first screened among the 651 *E. coli* strains isolated from water and sediments samples (**Table 3**). The prevalence of intestinal pathogenic strains was 1.99% (13/651) among which 0.30% (2/651) were STEC O174:H21 (*stx*2) and 0.61% (4/651) were EHEC O26:H11 (*eae, stx*1). A more detailed analysis of these intestinal pathogenic strains using phylotyping, qPCR microarray and targeted sequencing, showed a specific distribution along the continuum. No pathogenic strain was isolated from the upstream site close to the forest (Sébec). In contrast, EHEC and STEC were only isolated at the upstream pasture site (Selles). Two B1 phylogroup STEC O174:H21 (*stx*2) isolates having the same virulence gene profile (*astA, ihA, lpfA*_O113_, and *lpfA*_O26_) were isolated in water during both sampling campaigns. Four B1 phylogroup EHEC O26:H11 (*eae, stx*1) isolates were detected in sediments. At the Tourville – mixed urban and rural site – two E phylogroup atypical enteropathogenic *E. coli* (aEPEC) O153:H31 isolates with the same virulence gene profile were isolated from water and sediment. These aEPEC carry the *eae*-theta subtype, *espK* and some markers of the pathogenicity islands OI-57, OI-71 and OI-122 (Supplementary Table S2). MLST and RAPD analyses of these isolates confirmed the clonal identity of O174:H21, O26:H11 and O153:H31 isolates. At the downstream urban site in the Risle river, EAEC O99:H10 strain from A phylogroup and EAEC O111:H21 strain from B1 phylogroup were isolated from water and sediment, showed different virulence profiles (Supplementary Table S2). A DAEC O10:H4 strain (A phylogroup) and two aEPEC strains from serotype O81:H6 (B2 phylogroup unassignable) and O88:H25 (B1 phylogroup) were isolated from water. None of these strains was resistant to the 17 antibiotics tested except for two strains isolated from the downstream site: aEPEC resistant to chloramphenicol, and EAEC resistant to amoxicillin, amoxicillin + clavulanic acid, and cephalothin.

### Input of Shiga Toxin-producing *E. coli* (STEC and EHEC) Released by Cattle Farming at the Downstream Part of the Continuum

Shiga toxin-producing *E. coli* and EHEC were not detected from water samples in the downstream part of the rural continuum. Therefore, it should be possible to assess the contribution of the Selles River waterbodies to the total contamination of the water by Shiga toxin-producing *E. coli* at the upstream sites. Assuming that these pathogenic *E. coli* were only released in water at the upstream pasture site (Selles), it should be possible to estimate an order of magnitude of the transfer of STEC/EHEC released in water from the upstream site (Selles), to the downstream sites (Tourville and Risle), taking into account the residence time, the dilution of the water mass between the stations (**Table 1**), and the bacterial decay rate. For this purpose, two decay rates were taken into account for the intestinal pathogenic *E. coli*. One was obtained from an experimental approach (Berthe et al., 2013) used in this study. In a water microcosm, the two STEC harboring *stx*2 genes (EC 4199, EC 6086) and one EHEC harboring an *stx*1 gene (EC 6089) showed a decay rate of their culturable state of 6.8 10^-3^ h^-1^ when incubated in the dark in sterile estuarine water at 10°C (Berthe et al., 2013). However, because this estimation does not take into account the decay rate of *E. coli* due to predation, viral lysis, competition with autochthonous microorganisms, photolysis, and autolysis, we also used the decay rate estimated for fecal coliforms in rivers of the Seine watershed (45 10^-3^ h^-1^) at 20°C published by Servais et al. (2007).

At the upstream pasture site (Selles), the density of pathogenic intestinal *E. coli* present in the water column was estimated at 32 ± 4 *E. coli* CFU 100 mL^-1^, corresponding to 2% (2 STEC among the 99 *E. coli*) of the total number of *E. coli* 1.6 ± 0.3 10^3^ CFU 100 mL^-1^ (**Table 2**). Part of these intestinal pathogenic *E. coli* are attached to particles while a part is free-living cells. Taking into account the relationship between the percentage of attached *E. coli* in river waters, the concentration of SPM (Garcia-Armisen and Servais, 2009), and the SPM measured at the upstream pasture site (Selles), the percentage of intestinal *E. coli* associated with particles at the Selles site should range from 55 to 75% (i.e., 7.6 ± 3.3 to 24 ± 4.5 CFU 100 mL^-1^) during the low-flow period. These attached *E. coli* are susceptible to settling on sediment storage areas along the stream during low-flow period.

During a rain event and the resulting high-flow period, the flash flood took 6 h to arrive from the upstream pasture site (Selles) to the downstream site (Risle). The dilution was equal to 20 at the downstream site (Risle) with regards to the upstream site (Selles). In these conditions, the putative input of pathogenic intestinal *E. coli* ranged from 0.67 ± 0.13 to 0.97 ± 0.17 CFU 100 mL^-1^ (decay rate, 45 10^-3^ h^-1^) to 0.84 ± 0.16 to 1.15 ± 0.0 CFU 100 mL^-1^ (decay rate, 6.8 10^-3^ h^-1^), depending on the association with particles. All these values can be doubled if we take into account the putative additional input of EHEC due to the resuspension of sediment in the high-flow period.

In contrast, during the dry period, the waterbodies transfer from the upstream pasture site (Selles) to the downstream site (Tourville) reached 12 h with a dilution equal to 6, followed by 10 h with a final dilution equal to 40 up to the downstream Risle site (**Table 1**). In these hydrological conditions, input of Shiga toxin-producing *E. coli* from the upstream pasture site (Selles) to the main river (Risle) ranged from 0.03 ± 0.01 CFU 100 mL^-1^ to 0.04 ± 0.01 CFU 100 mL^-1^ (decay rate, 45 10^-3^ h^-1^) to 0.05 ± 0.00 to 0.07 ± 0.0 CFU 100 mL^-1^ (decay rate, 6.8 10^-3^ h^-1^), depending on the association with particles.

## Discussion

### Abundance and Origin of *E. coli* Contamination along a Small Rural Continuum with Cattle Farming

The aim of this study was to investigate the diversity of the *E. coli* population. It focused on the occurrence of pathogenic *E. coli* in flowing surface water draining a typical rural catchment. The land use of the three upstream sub-watersheds is characterized by smallholder cattles farming – for which the number of cattle is fewer than 50 – which corresponds to 64 to 70% of cattle farming in France (French Observatory of cattle industry, data 2012). The small rural continuum studied here is characterized by two opposite density gradients of animals (cattle and wild animals) and human populations. Along the continuum, based on the abundance of *E. coli*, the microbiological quality of the water was found to be of good to average quality according to the French water index (SEQ values, 2 10^2^ CFU 100 mL^-1^, to 2 10^3^ CFU 100 mL^-1^, respectively) established by the French Ministry of Environment and Regional Water Agencies, as well the WHO recommendations (WHO, 2011). In larger river drainage basins characterized by multiple watersheds with mixed agricultural land use, a correlation between *E. coli* density and the stream order has been reported (Seurinck et al., 2005; Mauffret et al., 2012). In stream water, a strong land use dependency has been shown between densities of *E. coli* and forest or urban areas (DiDonato et al., 2009). In contrast, here we showed a small increase of the abundance of bacterial fecal indicators in water along the rural continuum. This is mainly due to (i) an increase in human density, which is counterbalanced by the decrease in cattle density from upstream to downstream, and (ii) the dilution of this contamination along the hydrological network, combined with improved efficiency of the human wastewater treatments in the downstream part of the continuum studied.

In this study, the observation made on *E. coli* and *Enterococcus* and host-associated *Bacteroidales* markers are totally in agreement. As previously described in a watershed with intensive livestock rearing in France (Brittany) (Mauffret et al., 2012), abundance of both human and ruminant-associated *Bacteroidales* markers indicates a fecal contamination originating from ruminants at upstream rural sites (Selles and Sébec sites), and from humans at downstream more urbanized sites (Tourville and Risle).

At the upstream part, the low abundance of antibiotic-resistant bacteria could be explained by the low number of cattle receiving antibiotic therapy. Moreover, it should be noted that the administration of antibiotics to farm animals, as a growth promoter, was banned according to the European regulation. In contrast, the higher number of both *E. coli* multi-resistant to antibiotics and strains harboring class 1 integron reflected the human contamination in water at downstream urban sites. They also highlight the role of urban treatment plants in the spread of antibiotic resistant bacteria and genes in water environment (Oberlé et al., 2012; Rizzo et al., 2013).

### Spatial Change of Distribution of Phylogroup Distribution in Water

Despite similar land use and hydrological conditions, here we showed a spatial change of the phylogroup distribution within the *E. coli* population in both water and superficial sediments. At the upstream site close to a forest, higher abundances of *Escherichia* clade and of B2 phylogroup strains were observed. *Escherichia* clade strains are more frequently found in animals than in humans (Clermont et al., 2011). Furthermore, careful analysis of the B2 phylogroup strains showed that 50% of the B2 strains from the Sébec site were unassignable-O non-typable. It is actually a fact frequently observed in wild animal isolates (Smati et al., 2015). Only 19% were, however, unassignable-O non-typable at the downstream urban site (Risle). At the upstream pasture site, a greater proportion of both E and B1 phylogroups was detected, suggesting a fecal contamination of mainly bovine origin (Smati et al., 2015; Mercat et al., 2016). Conversely, in the two more urbanized sampling sites, A, D, and F phylogroups were more abundant, consistent with a higher human density (Massot et al., 2016). It has been demonstrated that the *E. coli* population structure in humans significantly differs from that observed in herbivorous animals such as cows (Carlos et al., 2010), even if some dominant strains could be shared between hosts in contact (Mercat et al., 2016). In a stream flowing in a small pasture watershed (France), or in estuarine water (Thames river, Canada) collected downstream from a rural landscape, it has been reported that B1 phylogroups were predominant while the A phylogroup was less abundant (Hamelin et al., 2007; Ratajczak et al., 2010; Berthe et al., 2013). This distribution of the phylogroups along the continuum argues for *E. coli* originating mainly from wild animals and cattle at the two upstream rural sites (Sébec and Selles), and from humans at the two downstream more urbanized sites (Tourville and Risle). Thus, along a hydrological network, like the rural continuum studied here, the structure of the *E. coli* population of the stream water from pasture land is strongly modified when this stream flows into a main river impacted by human sewage. To our knowledge, it was the first time that a strong spatial modification of the structure of the *E. coli* population was observed in surface water along such a small hydrological network (Sébec to Risle: 8 km/Selles to Risle: 5.2 km). Indeed, in greater watersheds, comparison of the genetic diversity of the *E. coli* populations sampled in contrasting water ecosystems have shown a spatial and temporal variation of the genetic diversity of *E. coli*, based on DNA fingerprinting or phylogroup distribution. Thus, it has been reported that in water the structure of the *E. coli* population was related to the hydrological conditions, the vicinity of the source of contamination, the stream order, and the land use (Hamelin et al., 2007; Lyautey et al., 2010). All of these abiotic parameters, combined with the distinct survival abilities, shape the diversity of the *E. coli* population in the water environment (Van Elsas et al., 2011; Berthe et al., 2013).

### Prevalence of Pathogenic *E. coli*

In water and sediment of the rural continuum studied here, the prevalence of intestinal pathogenic *E. coli* was 1.99%, mainly belonging to the B1 phylogroup (8/13). However, this study shows a spatial distribution of pathogenic *E. coli* in surface water, over a 5.2 km distance downstream from the pasture site (Selles) to the urbanized site (Risle). At the upstream pasture site, while the selective chromogenic media specific of glucuronidase-positive *E. coli* used in this study did not allow the detection of *E. coli* O157:H7 (glucuronidase-negative strain), highly pathogenic STEC O174:H21 (*stx*2) (0.3%) and EHEC O26:H11 (*stx*1, *eae*) (0.61%), belonging to the B1 phylogroup were isolated in water and surficial sediments. In sediment, the four EHEC O26:H11 (*eae, stx*1) had similar virulence profiles showing the presence of genetic markers and pathogenicity islands characteristic of typical EHEC (Bugarel et al., 2011). This latter EHEC serotype was also isolated downstream an intensive livestock farming watershed in another French region, Brittany, more precisely in shellfish batch (Balière et al., 2016). One should note that atypical EHEC O174:H21 had already been reported in human hemolytic uraemic syndrome (HUS) cases (Zhang et al., 2014) and that EHEC O26:H11 predominates in reported HUS cases worldwide. Indeed, in 2005, in northwestern France where our study sites were located, EHEC O26:H11 were involved in a human outbreak due to raw milk cheese (INVS, 2007). Although STEC have been reported to be prevalent in wild animals living close to a cattle farm (Saxena et al., 2015), it is likely that these pathogenic *E. coli* came from the cattle reservoir. Indeed, in our study, no pathogenic strain was isolated in the site close to the forest (Sébec) while high abundance of unassignable B2 strains suggested the presence of *E. coli* from wild animals, mainly boar, roe and deer (Smati et al., 2015). In other studies, the prevalence in the surface water of STEC (*stx*) or EHEC (*stx*+*eae*) ranged from absence in a Japanese river (Gomi et al., 2015), to 14 and 1.8% in a lake in Canada (Chandran and Mazumder, 2015), and 11% in a subtropical watershed in Australia (Sidhu et al., 2013). However, the value reached 66% (*stx*1 or *stx*2) in a tributary of the Ganges (India) impacted by untreated human sewage (Ram et al., 2009). In comparison, the frequencies of STEC/EHEC reported in the treated effluent of a WWTP connected to a hospital and a slaughterhouse, or in the effluent of a slaughterhouse were 0.2 and 0.5%, respectively (Diallo et al., 2013; Um et al., 2016). The results obtained in sediments are consistent with the study reported by Balière et al. (2015), which showed the prevalence of STEC in coastal sediments (0.85%; 1/83 isolated *E. coli*) and in freshwater sediments (0.17%; 4/2,036 isolated *E. coli*) in a larger coastal watershed in Normandy (1000 km^2^). These results illustrated that river sediments could be a reservoir of pathogenic *E. coli*, where some strains can persist and then could be resuspended in the water column, mainly in high flow periods (Ouattara et al., 2011; Pachepsky and Shelton, 2011).

At the downstream more urbanized sites (Tourville and Risle), *E. coli* pathotypes traditionally associated with a human reservoir, i.e., aEPEC, EAEC, and DAEC, were isolated in water and sediments. At the mixed rural and urban site (Tourville), the *E. coli* (aEPEC) O153:H31 carried the *eae*-theta subtype and virulence genetic markers shared by EHEC, such as in particular *espK* and some markers of the pathogenicity islands OI-57, OI-71 and OI-122. This suggested a potential to cause hemorrhagic colitis after acquisition of Stx phages. At the downstream urban site (Risle), the *eae-*subtype of the two *E. coli* (aEPEC) O81:H6 and O88:H25 isolates was different from the four main *eae*-subtypes (gamma, beta, epsilon and theta) associated with the typical EHEC of the Top7 EHEC serogroups. However, it should be pointed out that O88:H25 isolates have already been isolated in human cases (Pedroso et al., 1993) and could potentially cause acute diarrhea in children. At the same site, enteroaggregative *E. coli* (EAEC) O99:H10 and EAEC O111:H21 were also detected from water and sediment, respectively. In Northern Ireland, the intra-intestinal pathogenic EAEC O111:H21 isolated in water was associated with human disease (Dallman et al., 2012). These intra-intestinal *E. coli* pathotypes were also detected in surface water impacted by human sewage or located in urban areas (Hamelin et al., 2007; Ramírez Castillo et al., 2013; Sidhu et al., 2013). Moreover, extra-intestinal pathogenic *E. coli* of D phylogroup, belonging to CGA, were mainly isolated in the downstream urban site (Risle). It highlighted the role of the wastewater effluent in the dissemination of this uropathogenic *E. coli* CGA in the environment (Boczek et al., 2007). As previously reported, pathogenic *E. coli* are persistent in sediment of stream river (Garzio-Hadzick et al., 2010; Haack et al., 2015), or freshwater lake (Chandran et al., 2011). Occurrence of intestinal pathogenic strains in sediments – mainly STEC or EHEC as shown here – underlines the role of this environment as a putative secondary reservoir of pathogenic strains that could be re-suspended in water in high-flow periods (Pachepsky and Shelton, 2011). However, no STEC or EHEC have been isolated 5.2 km downstream of the rural aquatic continuum. Yet, it has been demonstrated by microcosms and *in situ* experiments that STEC, such as *E. coli* O157:H7, better survive in water than commensal *E. coli*. This is due to a better resistance to both solar irradiation and predation (Jenkins et al., 2011; Williams et al., 2012). However, the estimated input of non-O157 STEC *E. coli –* released in water at the upstream pasture site – at the downstream site was low (<1 CFU 100 mL^-1^), likely due to dilution along the hydrologic network, predation, lysis, and loss of culturability.

The small number of sampling campaigns could constitute a potential limitation of this study. In the same way it would be interesting to extend this study after a stormwater during a dry period. Indeed, in these hydrological conditions, there is an important run-off of pasture land, combined with putative septic-tank overflow. However, the monitoring of the water bodies carried out here, allowed a close analysis of *E. coli* population in stream water, for similar land use and hydrological conditions. Along a rural continuum representative of the cattle farming watershed in France, while a small increase of the density of bacterial indicators of fecal contamination was observed, this study showed strong changes in the structure and antibiotic resistance of the *E. coli* population, both reflecting the land use and the stream order. Prevalence of pathogenic strains in water and sediment reflected the land use with presence of STEC/EHEC close to the upstream pasture site, while in the more urbanized downstream sites, aEPEC/EAEC, DAEC and extra-intestinal *E. coli* belonging to CGA of human origin were isolated. It should be noted that, in France, during the sampling period of this study (2009–2010), 238 Shiga toxin-producing *E. coli* (STEC) infections were observed in hospitals, among which O26 and O111 serogroups accounted for 4 and 1% of the infections, respectively (ANSES, 2010). These results highlight the role of both the runoff of pasture land and the wastewater effluent, in the dissemination of pathogenic *E. coli* from human or bovine origins in surface water. This study shows that change of distribution phylogroups within *E. coli* population reflected the use of the watershed. These results highlight two paradoxes: (i) in a stream draining a rural watershed, characterized by small cattle farming, highly pathogenic *E. coli* could be isolated in water while microbial quality of the water was good or to average quality according to the French water index; (ii) while loss of STEC was observed along the hydrological network these strains could persist in sediment. This underlines the role of sediment as a secondary reservoir of fecal pathogens, which could be resuspended later in the water column (Droppo et al., 2009). Thus, to improve waterborne pathogen surveillance, besides the only monitoring of both *E. coli* and *Enterococcus*, it could be necessary to monitor STEC released in water by small cattle farming especially following run-off events.

## Author Contributions

The work presented here was carried out in collaboration with all authors. FP, TB, and ED defined the research theme. FP, TB, KO, and MF defined sampling strategy and designed methods and experiments. TB and KO carried out the laboratory experiments, strains isolation and antibiotic resistance analysis, ED and OC carried out the phylotyping of *E. coli*, MG and KO carried out the quantification of *Bacteroidales* markers, and SD and PF carried out the characterisation of the virulence genes and additional molecular serotyping. MF carried out the statistical analysis. TB, PS, ED, SD, PF, and FP analyzed the data, interpreted the results and FP wrote the paper. All authors have contributed to, seen and approved the final manuscript.

## Conflict of Interest Statement

The authors declare that the research was conducted in the absence of any commercial or financial relationships that could be construed as a potential conflict of interest. The reviewer AFFR and handling Editor declared their shared affiliation and the handling Editor states that the process nevertheless met the standards of a fair and objective review.

## Footnotes

1 http://agreste.agriculture.gouv.fr

2 http://services.meteofrance.com/

3 http://www.sfm-microbiologie.org/
