# Supplementary material for: Change in the Structure of Escherichia coli Population and the Pattern of Virulence Genes along a Rural Aquatic Continuum
Source: Front Microbiol. 2017 Apr 18;8:609. doi: 10.3389/fmicb.2017.00609 (PMC5394106; doi:10.3389/fmicb.2017.00609)
Supplement: Supplementary file 1 [file Table_1.PDF]

Table S1: Diversity of B2 *E. coli* isolates along the rural continuum

Site	Sample	N	Subgroup [a]	O type [b]
Sébec (*n*=42)				
	Water (*n*=25)	3	I	O18
		1	I	O83
		1	I	O7
		1	I	ONT [c]
		1	X	ONT
		1	UA [d]	O83
		17	UA	ONT
	Sediment (*n*=17)	7	IV	O2b
		6	X	ONT
		4	UA	ONT
Selles (*n*=7)				
	Water (*n*=4)	1	II	ONT
		1	UA	O6
		1	UA	O83
		1	UA	ONT
	Sediment (*n*=3)	1	IX	O2b
		1	X	ONT
		1	UA	ONT
Tourville (*n*=12)				
	Water (*n*=5)	1	I	O21
		1	UA	O83
		1	UA	O2b
		2	UA	ONT
	Sediment (*n*=7)	1	IV	O2b
		3	VI	O22
		1	VII	O75
		2	UA	O2b
Risle (*n*=31)				
	Water (*n*=18)	**2** [e]	**I**	**O25b**
		1	I	O18
		1	I	ONT
		**1**	**II**	**O18**
		**3**	**III**	**O6**
		1	IV	O83
		**2**	**VI**	**O4**
		**1**	**IX**	**O1**
		1	UA	O81
		2	UA	O75
		3	UA	ONT
	Sediment (*n*=13)	1	I	O6
		**1**	**III**	**O6**
		3	IV	O2b
		1	VII	O75
		**3**	**IX**	**O1**
		**1**	**IX**	**O18**
		3	UA	ONT

[a] Determined as in (Clermont et al., 2014); [b] Determined as in (Clermont et al., 2007); [c] ONT: O-non typable; [d] UA unassigned; [e] Typical human ExPEC clones are indicated in bold
