# Supplementary material for: Change in the Structure of Escherichia coli Population and the Pattern of Virulence Genes along a Rural Aquatic Continuum
Source: Front Microbiol. 2017 Apr 18;8:609. doi: 10.3389/fmicb.2017.00609 (PMC5394106; doi:10.3389/fmicb.2017.00609)
Supplement: Supplementary file 2 [file Table_2.PDF]

Table S2: Virulence gene profile of *E. coli* isolated from water and sediment

Sampled site / *E. coli* isolate / Virulence gene		Selles						Tourville		Risle				
		Water		Sediment				Water	Sediment	Water				Sediment
		EC 4199	EC 6086	EC 6089	EC 6096	EC 6111	EC 6115	EC 6230	EC 6316	EC 4312	EC 4321	EC 4330	EC 6138	EC 4342
*stx*1	*stx*1	-	-	+	+	+	+	-	-	-	-	-	-	-
	VT1	-	-	+	+	+	+	-	-	-		-	-	
	-	-	-	+	+	+	+	-	-	-	-	-	-	-
*stx*2	*stx*2	+	+	-	-	-	-	-	-	-	-	-	-	-
	VT2	+	+	-	-	-	-	-	-	-	-	-	-	-
	*stx*2c-8	+	+	-	-	-	-	-	-	-	-	-	-	-
	*stx*2dg	+	+	-	-	-	-	-	-	-	-	-	-	-
	*stx*2d2	+	+	-	-	-	-	-	-	-	-	-	-	-
	*stx*2d-global	+	+	-	-	-	-	-	-	-	-	-	-	-
eae	*eae*	-	-	+	+	+	+	+	+	-	-	+	+	-
	eae-beta	-	-	+	+	+	+	-	-	-	-	-	-	-
	eae-theta	-	-	-	-	-	-	+	+	-	-	-	-	-
Serotype	*wzx*O174	+	+	-	-	-	-	-	-	-	-	-	-	-
	*wzx*O81			-	-	-	-	-	-	-	-		-	-
	H21	+	+	-	-	-	-	-	-	-	-	-	-	
	H25			-	-	-	-	-	-	-	-	-		-
EAEC	*agg*R	-	-	-	-	-	-	-	-	+	-	-	-	+
	*aat*A	-	-	-	-	-	-	-	-	+	-	-	-	+
	aap	-	-	-	-	-	-	-	-	+	-	-	-	+
	pic	-	-	-	-	-	-	-	-	+	-	-	-	+
	*set*1	-	-	-	-	-	-	-	-	+	-	-	-	+
HPI	*irp*2	-	-	+	+	+	+	-	-	+	+	+	-	+
	*fyu*A	-	-	+	+	+	+	-	-	+	+	+	-	+
Heat-stable enterotoxin	*ast*A	+	+	+	+	+	+	-	-	-	-	+	-	+
OI-15	*eha*A	+	+	+	+	+	+	+	+	-	-	-	+	+
OI-43	Z1151	-	-	+	+	+	+	-	-	-	-	-	-	-
	Z1153	-	-	+	+	+	+	-	-	-	-	-	-	-
	Z1155	-	-	+	+	+	+	-	-	-	-	-	-	-
	Z1156	-	-	+	+	+	+	-	-	-	+	-	-	-
OI-43 / OI-48	*iha*	+	+	+	+	+	+	-	-	+	-	-	-	+
	*ter*E	-	-	+	+	+	+	-	-	+	-	-	-	-
	*ure*D	-	-	+	+	+	+	-	-	-	-	-	-	-
OI-44	*esp*V	-	-	+	+	+	+	-	-	-	-	-	-	-
OI-50	*esp*K	-	-	+	+	+	+	+	+	-	-	-	-	-
	*esp*N	-	-	+	+	+	+	-	-	-	-	-	-	-
	*esp*X7	-	-	+	+	+	+	-	-	-	-	-	-	-
OI-57	Z2096	-	-	+	+	+	+	-	-	-	-	-	-	-

	**Z2098**	-	-	+	+	+	+	-	-	-	-	-	-	-
	**Z2099**	-	-	+	+	+	+	-	-	-	-	-	-	-
	**Z2121**	-	-	+	+	+	+	+	+	-	-	-	+	-
	***ecs*1763**	-	-	+	+	+	+	-	-	-	-	-	-	-
**OI-71**	***nle*F**	-	-	+	+	+	+	+	+	-	-	-	-	-
	***nle*G**	-	-	+	+	+	+	-	-	-	-	-	-	-
	***esp*M1**	-	-	+	+	+	+	-	-	-	-	-	-	-
	***nle*H1-2**	-	-	+	+	+	+	+	+	-	-	+	+	-
	***nle*A**	-	-	+	+	+	+	-	-	-	-	-	-	-
	***ecs*1822**	-	-	+	+	+	+	-	-	-	-	-	-	-
**OI-108**	***esp*M2**	-	-	+	+	+	+	-	-	-	-	-	-	-
	***esp*W**	-	-	-	+	+	+	-	-	-	-	-	-	-
**OI-122**	***ent***	-	-	+	+	+	+	+	+	-	-	-	-	-
	***nle*B**	-	-	+	+	+	+	+	+	-	-	-	-	-
	***nle*E**	-	-	+	+	+	+	+	+	-	-	-	-	-
	**Z4331**	-	-	+	+	+	+	-	-	-	-	-	-	-
	***efa*1**	-	-	+	+	+	+	-	-	-	-	-	-	-
	***efa*2**	-	-	+	+	+	+	-	-	-	-	-	-	-
**pO157**	***ehx*A**	-	-	+	+	+	+	-	-	-	-	-	-	-
	***ecf*1**	-	-	+	+	+	+	-	-	-	-	-	-	-
	***tox*B**	-	-	+	+	+	+	-	-	-	-	-	-	-
	***kat*P**	-	-	+	+	+	+	-	-	-	-	-	-	-
	**espP**	-	-	+	+	+	+	-	-	-	-	-	-	-
**Long polar fimbriae**	***lpf*AO113**	+	+	+	+	+	+	-	-	-	-	-	+	+
	***lpf*AO157**	-	-	-	-	-	-	+	+	-	-	-	-	-
	***lpf*AO26**	ND	+	+	+	+	+	-	-	-	-	-	+	-
**Bundle-forming pilus**	***bfp*A**	-	-	-	-	-	-	-	-	-	-	-	+	-
