# Supplementary material for: Change in the Structure of Escherichia coli Population and the Pattern of Virulence Genes along a Rural Aquatic Continuum
Source: Front Microbiol. 2017 Apr 18;8:609. doi: 10.3389/fmicb.2017.00609 (PMC5394106; doi:10.3389/fmicb.2017.00609)
Supplement: Supplementary file 3 [file Table_3.PDF]

Table S3: Phylogroup distribution and antibiotic resistance of *E. coli* populations along a rural continuum

Site			Selles			Sébec			Tourville			Risle		
			Water	Sediment	Total	Water	Sediment	Total	Water	Sediment	Total	Water	Sediment	Total
Isolate number			99	49	148	99	59	158	96	59	155	94	96	190
Phylogroup N (%)	A		16 (16)	7 (14)	23	15 (15)	4 (6.7)	19	34 (35.4)	9 (15.25)	43	33 (35,5)	29 (30,0)	62
	B1		49 (49)	22 (44)	71	31 (31)	11(18.3)	42	35 (36.4)	24 (40.7)	59	22 (23.7)	33 (34.0)	55
	B2		4 (4)	4 (8)	8	24 (24)	18 (30)	42	5 (5.2)	8 (13.6)	13	18 (19.35)	13 (13.5)	31
	C		0	1 (2)	1	0	0	0	1 (1.0)	0	1	4 (4.3)	1 (1.0)	5
	D	Non CGA	8 (8)	0	0	0	0	0	4 (4.0)	4 (6.8)	8	8 (8.6)	3(3.0)	11
		CGA [a]	1 (1)	3 (6)	4	3 (3)	2 (3.3)	5	1 (1)	0	1	4 (4.3)	9 (9.3)	13
	E		14 (14)	6 (12)	20	4 (4)	1 (1.7)	5	5 (5.2)	2 (3.4)	7	0	1 (1.0)	1
	F		2 (2)	1 (2)	3	0	0	0	2 (2.0)	2 (3.4)	4	4 (4.3)	2 (2.1)	6
	Clade (II, III, V)		5 (0)	5 (10)	10	22 (22)	23 (38.3)	45	9 (9.3)	10 (17)	19	1 (1)	5 (5.2)	6
Antibiotic resistance N (%)	No resistance		93 (93)	48 (98)	141	79 (79)	55 (95)	134	80 (83.3)	56 (95)	136	63 (64.5)	74 (80)	137
	1-3		5 (5)	1 (2)	6	14 (14)	3 (5)	17	7 (7.3)	3 (5)	10	13 (14)	8 (8.5)	21
	> 3		1 (1)	0	1	7 (7)	0	7	9 (9.3)	0	9	18 (19.3)	14 (14.5)	32
	Integrase gene *intI*1		1	0	1	1	0	1	5	0	5	5	0	5

[a] CGA:clonal group A
